# Equilibrium space and a pseudo linearization of nonlinear systems

**DOI:** 10.1038/s41598-022-25616-1

**Published:** 2022-12-07

**Authors:** Ryotaro Sakata, Tatsuya Oshima, Shin Kawai, Triet Nguyen-Van

**Affiliations:** 1 Department of Intelligent and Mechanical Interaction Systems, University of Tsukuba, Tsukuba, 305-8573 Japan; 2 Present Address: Electronic Control and Simulation Group, Toyota Systems Corporation, Nagoya, Japan

**Keywords:** Electrical and electronic engineering, Mechanical engineering, Aerospace engineering

## Abstract

This paper attempts to extend the concept of the equilibrium point to what is called equilibrium space, which can adapt to a system in which there exists an infinite number of equilibrium points. In the context of Lyapunov’s linearization method extended for the equilibrium space, this paper proposes a pseudo linearization, from which we can derive a linear representation for a nonlinear system. The equilibrium state of this pseudo linearization and its stability are shown to be the same as that of the original nonlinear system. As an example of the applicability, the proposed pseudo linearization is applied to derive a discrete-time model for a control moment gyroscope system from a nonlinear continuous-time model. Simulation results show that the discrete-time model derived using the proposed pseudo linearization yields responses that are closer to that of the continuous-time model than the discrete-time model derived by the well-known forward-difference method and the conventional pseudo linear representation method, even with a large sampling interval.

## Introduction

Most engineering systems based on natural phenomena are nonlinear. Analyzing the stability and designing controllers for nonlinear systems are important issues in system control theory^1^. However, despite the pioneering research in this field, there is no universal method for designing nonlinear control systems^2^. To study the stability of nonlinear systems, the Lyapunov theory that includes both the direct method and linearization method (or indirect method) is one of the most general and useful approaches. The direct method is used to study the global stability of nonlinear systems using the Lyapunov function; however, a drawback of this is that there is no general way of deducing the Lyapunov function for a specific system. By contrast, the linearization method studies the local stability around an equilibrium point based on its linear approximation, and this has become an important tool for designing controllers for nonlinear systems using the well-known linear control theories^3–5^. In recent years, Koopman operator and contraction analysis have become two of the popular approaches to analyze the stability of singly hyperbolic equilibria of nonlinear systems globally and exactly by way of linear system theory^6,7^.

In systems such as a robot moving on a horizontal plane without friction^8,9^ or a pendulum not under the influence of gravity^10,11^, in which the position/angle and velocity are selected as state variables and the initial velocity is set to zero, for an arbitrary initial position, the system remains in the initial state for all future instances of time. More specifically, these systems have an infinite number of equilibrium points, which are independent of the position. Such a set of non-isolated equilibria is known as a manifold of equilibria^12,13^. It should be noted that this concept of manifold of equilibria is different from the center manifold of an isolated equilibirum^14,15^. Lyapunov’s linearization method is a useful tool to investigate the stability of a single equilibrium point. However, for systems which have an infinite number of equilibrium points, it is not realistic to investigate all equilibrium points individually. Furthermore, the above-mentioned examples are known as nonholonomic systems, and the linearization may change the controllability of the original nonlinear system^16–19^. Therefore, even though the nonlinear system is controllable, its linearization becomes uncontrollable and is unattainable for controller design. This paper attempts to extend the concept of equilibrium points to what is called equilibrium space, which can adapt to systems that have an infinite number of equilibrium points. In the context of Lyapunov’s linearization method, this paper proposes pseudo linearization, by which we can derive a nonlinear system presented by the linear form^20–23^. The main contributions of this paper are as stated as follows:

- Proposing a definition of equilibrium space, which is an extension of the concept of the equilibrium point;
- Proposing a pseudo linearization based on the equilibrium space, and showing that the equilibrium state of this pseudo linearization and its stability are the same as that of the original nonlinear system;

The proposed pseudo linearization is applied to derive a discrete-time model for a control moment gyroscope (CMG) system, an application of the gyro effect, which is often used as an attitude control actuator for artificial satellites and spacecraft^24–27^. The remainder of this paper is organized as follows: “Equilibrium point and Lyapunov’ linearization” section summarizes the definition of equilibrium points and Lyapunov’s linearization method. The definition of equilibrium space, the correspondent pseudo linearization, and their properties are presented in “Equilibrium space and pseudo linearization” section. An application of pseudo linearization in deriving a discrete-time model of the CMG system is presented in “Discrete-time model of CMG system based on pseudo linearization” section. Simulation results for the CMG system are presented in “Simulations” section, and finally, conclusions are given in “Conclusion” section.

## Equilibrium point and Lyapunov’ linearization

First, we consider a system described by the following state space equation

1 $$\begin{aligned} \dot{{\mathbf{x}}}\left( t \right) = {\mathbf{f}}\left( {\mathbf{x}}\left( t \right) \right) , \end{aligned}$$

where ${\mathbf{x}}$ is a system state, which belongs to a state space ${\mathbf{X}} \subset R^{n}$, time *t* is an independent variable, and ${\mathbf{f}} :{\mathbf{X}} \rightarrow R^{n}$ is a continuously differentiable system function.

Suppose ${\mathbf{x}}_{ep} \in {\mathbf{X}}$ is an equilibrium point of (1), i.e.,

2 $$\begin{aligned} {\mathbf{f}}\left( {\mathbf{x}}_{ep} \right) = {\mathbf{0}}. \end{aligned}$$

A linearization of (1) about the equilibrium point ${\mathbf{x}}_{ep}$ is given by

3 $$\begin{aligned} \dot{{\mathbf{x}}}\left( t \right) = D{\mathbf{f}}\left( {\mathbf{x}}_{ep} \right) \left( {\mathbf{x}}\left( t \right) - {\mathbf{x}}_{ep} \right) {\mathop {=}\limits ^{\text{def}}} {\mathbf{f}}_{lin}\left( {\mathbf{x}}\left( t \right) \right) , \end{aligned}$$

where $D{\mathbf{f}}\left( {\mathbf{x}}_{ep} \right)$ is a Jacobian matrix of ${\mathbf{f}}\left( {\mathbf{x}} \right)$ at ${\mathbf{x}}_{ep}$, i.e.,

4 $$\begin{aligned} D{\mathbf{f}}\left( {\mathbf{x}}_{ep} \right) = \left. \frac{\partial {\mathbf{f}}\left( {\mathbf{x}} \right) }{\partial {\mathbf{x}}} \right| _{{\mathbf{x}} = {\mathbf{x}}_{ep}}. \end{aligned}$$

It should be noted that

5 $$\begin{aligned} {\mathbf{f}}\left( {\mathbf{x}}_{ep} \right) = {\mathbf{f}}_{lin}\left( {\mathbf{x}}_{ep} \right) = {\mathbf{0}}. \end{aligned}$$

Thus, if ${\mathbf{x}}_{ep}$ is an equilibrium point of the system described through Eq. (1), it is also an equilibrium point of the linearized system described by Eq. (3). Furthermore, we have

6 $$\begin{aligned} D{\mathbf{f}}_{lin}\left( {\mathbf{x}}_{ep} \right) = D{\mathbf{f}}\left( {\mathbf{x}}_{ep} \right) . \end{aligned}$$

Following the Lyapunov’s indirect theorem, the stability of ${\mathbf{x}}_{ep}$ in the original system and the linearized system are identical locally^4,5^.

## Equilibrium space and pseudo linearization

The above-mentioned Lyapunov’s indirect theorem is a useful tool for investigating the stability of a single equilibrium point. However, with a system containing an infinite number of equilibrium points, investigating all equilibrium points individually is unrealistic. In this section, the concept of an equilibrium point and the corresponding linearization presented in “Equilibrium point and Lyapunov’ linearization” section are extended to develop a concept for equilibrium space and a pseudo linearization.

### Equilibrium space

#### **Definition 1**

(*Equilibrium space*) For a system described by Eq. (1), there is a subspace ${\mathbf{X}}_{es}$ of the state space ${\mathbf{X}}$ (${\mathbf{X}}_{es} \subset {\mathbf{X}}$), satisfying

7 $$\begin{aligned} {\mathbf{X}}_{es} = \left\{ {\mathbf{x}}_{es}\left( t \right) \left| {\mathbf{f}}\left( {{\mathbf{x}}_{es}\left( t \right) }\right) = {\mathbf{0}}\right. \right\} , \end{aligned}$$

where

8 $$\begin{aligned} {{\mathbf{x}}_{es}\left( t \right) = {\mathbf{Tx}}\left( t \right) + \left( {{{\mathbf{I}}} - {{\mathbf{T}}}} \right) {{\mathbf{\chi }}_{es}}} \end{aligned}$$

and ${\mathbf{I}}$ is an $n \times n$ identity matrix, ${{\mathbf{T}}} \in {R^{n \times n}}$ is a diagonal matrix, whose elements are one or zero, and has a rank of *m* ($m \le n$), and $\mathbf{\chi }_{es} \in {\mathbf{X}}$ represents state values, in which case ${\mathbf{X}}_{es}$ and ${\mathbf{x}}_{es}$ are the equilibrium space and equilibrium state of (1), respectively. The parameter *m* is called an order of the equilibrium space ${\mathbf{x}}_{es}$. The $\left( {n - m} \right)$-dimensional manifold corresponding to the nonzero element of the matrix $\left( {\mathbf{I}} - {\mathbf{T}} \right)$ is known as a manifold of equilibria^12^.

It should be noted that if there exists an ${\mathbf{x}}_{es}$ such that ${\mathbf{f}}\left( {{\mathbf{x}}_{es}} \right) = {\mathbf{0}}$, then ${\mathbf{x}}_{es}$ always can be expressed by the form of (8), which is composed of determined states $({\mathbf{I}} - {\mathbf{T}} ){{\mathbf{\chi }}_{es}}$ and undetermined states $\mathbf{Tx}(t)$. The matrix ${\mathbf{T}}$ and vector ${\mathbf{\chi }}_{es}$ are unique.

#### Example 1

The equilibrium space of the following system

9 $$\begin{aligned} \begin{array}{l} {\dot{x}}_{1}\left( t \right) = x_{1}\left( t \right) - 2,\\ {\dot{x}}_{2}\left( t \right) = \left( x_{1}\left( t \right) + x_{2}\left( t \right) \right) \left( x_{1}\left( t \right) - 2 \right) , \end{array} \end{aligned}$$

is given by

10 $$\begin{aligned} {\mathbf{x}}_{es}\left( t \right) = \left[ {\begin{array}{*{20}{c}} 0&{}\quad 0\\ 0&{}\quad 1 \end{array}} \right] \left[ {\begin{array}{*{20}{c}} {{x_1}\left( t \right) }\\ {{x_2}\left( t \right) } \end{array}} \right] + \left[ {\begin{array}{*{20}{c}} 1&{}\quad 0\\ 0&{}\quad 0 \end{array}} \right] \left[ {\begin{array}{*{20}{c}} 2\\ 0 \end{array}} \right] = \left[ {\begin{array}{*{20}{c}} 2\\ {{x_2}\left( t \right) } \end{array}} \right] , \end{aligned}$$

and has the order of $m=1$. The manifold of equilibria of this system is the line $x_1=2$.

#### Remark 1

For an arbitrary point ${\mathbf{x}}_{ep} \in {\mathbf{X}}_{es}$, ${\mathbf{x}}_{ep}$ is an equilibrium point of (1). The equilibrium space ${\mathbf{X}}_{es}$ is composed of an infinite number of equilibrium points.

#### Remark 2

When the order of the equilibrium space is zero ($m=0$), i.e., ${\mathbf{T}}={\mathbf{0}}$, the equilibrium space is a set of conventionally isolated equilibrium points ${\mathbf{\chi }}_{es}$.

#### Remark 3

When the order of the equilibrium space $m=n$, i.e., ${\mathbf{T}}={\mathbf{I}}$, the equilibrium space ${\mathbf{X}}_{es}$ and the state space ${\mathbf{X}}$ are identical. In other words, system (1) is not dynamic, i.e., it is a static system.

#### Remark 4

For a linear system $\dot{{\mathbf{x}}} = {\mathbf{Ax}}$, the order of the equilibrium space *m* is identical to the dimension of the null space of the system matrix *A*.

### Pseudo linearization based on equilibrium space

In the context of the equilibrium point presented in “Equilibrium point and Lyapunov’ linearization” section, we can derive the following results for the equilibrium space.

By linearizing system (1) about ${\mathbf{x}}_{es}$ , we can derive the following system

11 $$\begin{aligned} \dot{{\mathbf{x}}} = D{\mathbf{f}}\left( {{\mathbf{x}}_{es}} \right) \left( {{\mathbf{x}} - {\mathbf{x}}_{es}} \right) {\mathop {=}\limits ^{\text{def}}} {{\mathbf{f}}_{es}}\left( {\mathbf{x}} \right) . \end{aligned}$$

It should be noted that since the equilibrium state ${\mathbf{x}}_{es}$ is composed of part of the state ${\mathbf{x}}$, although the system given by Eq. (11) is represented by a linear form, it is a nonlinear system, and this is known as a pseudo-linear representation. While the conventional pseudo linear form usually represents the original nonlinear function ${\mathbf{f}}$ in a linear form^20–23^, i.e.,

12 $$\begin{aligned} \dot{{\mathbf{x}}} = {\mathbf{f}}\left( {\mathbf{x}} \right) = {{\mathbf{A}}}\left( {\mathbf{x}} \right) {\mathbf{x}} + {{\mathbf{b}}}, \end{aligned}$$

the pseudo-linear system (11) is an approximation of the original system. By presenting a nonlinear system by the pseudo-linear form, theories of linear system can be applied to analyse or design controllers for the nonlinear system^20,23,28^. Additionally, there are some characteristic properties, which are extensions of that of the equilibrium point, as detailed below.

#### Example 2

Consider a system same as the one used in Example 1. The conventional pseudo-linear representation of this system is given by Eq. (12), where $${\mathbf{x}} = {\left[ {\begin{array}{*{20}{c}}{{x_1}}&{{x_2}}\end{array}} \right] ^T}$$, $${{\mathbf{b}}} = {\left[ {\begin{array}{*{20}{c}}{ - 2}&0\end{array}} \right] ^T}$$, and

13 $$\begin{aligned} {{\mathbf{A}}}\left( {\mathbf{x}} \right) = \left[ {\begin{array}{*{20}{c}} 1&{}\quad 0\\ {{x_1} - 2}&{}\quad {{x_1} - 2} \end{array}} \right] . \end{aligned}$$

It should be note that the matrix ${\mathbf{A}}({\mathbf{x}})$ may take a different form, i.e.,

14 $$\begin{aligned} {{\mathbf{A}}}\left( {\mathbf{x}} \right) = \left[ {\begin{array}{*{20}{c}} 1&{}\quad 0\\ {{x_1} + {x_2} - 2}&{}\quad { - 2} \end{array}} \right] . \end{aligned}$$

The pseudo-linear approximated system about the equilibrium state ${\mathbf{x}}_{es}$ as given by (10) can be uniquely written in the form of (11), where

15 $$\begin{aligned} D{\mathbf{f}}\left( {{\mathbf{x}}_{es}} \right) = \left[ {\begin{array}{*{20}{c}} 1&{}\quad 0\\ {2{x_1} + {x_2} - 2}&{}\quad {{x_1} - 2} \end{array}} \right] . \end{aligned}$$

#### Theorem 1

If ${\mathbf{x}}_{es}$ is an equilibrium state of the system represented by Eq. (1), then it is also an equilibrium state of the pseudo linearized system as represented by Eq. (11).

#### Proof

From the definition of equilibrium space, and after substituting ${\mathbf{x}}_{es}$ to ${\mathbf{f}}_{es}$ given by Eq. (11), we have

16 $$\begin{aligned} {\mathbf{f}}\left( {{\mathbf{x}}_{es}} \right) = {{\mathbf{f}}_{es}}\left( {{\mathbf{x}}_{es}} \right) = {\mathbf{0}}. \end{aligned}$$

Thus, proof of theorem 1 is provided. $\square$

#### Theorem 2

The stabilities of ${\mathbf{x}}_{es}$ in the original system (1) and the pseudo linearized system (11) are identical locally.

Before proving theorem 2, we show the result of the following lemma.

#### Lemma 1

If ${\mathbf{x}}_{es} \in {\mathbf{X}}_{es}$, then the Jacobian matrix of ${\mathbf{f}}$ at ${\mathbf{x}}_{es}$ and the matrix ${\mathbf{T}}$ in definition 1 are orthogonal, i.e.,

17 $$\begin{aligned} D{\mathbf{f}}\left( {{\mathbf{x}}_{es}} \right) {{\mathbf{T}}} = {\mathbf{0}}. \end{aligned}$$

#### Proof

For ${\mathbf{x}}_{es} \in {\mathbf{X}}_{es}$, we have

18 $$\begin{aligned} {\mathbf{f}}\left( {{\mathbf{x}}_{es}} \right) = {\mathbf{0}}. \end{aligned}$$

By noting that ${\mathbf{x}}_{es}$ is composed of ${\mathbf{x}}$, we can consider ${\mathbf{f}}\left( {{\mathbf{x}}_{es}} \right)$ as a function of ${\mathbf{x}}$. Differentiating both sides of Eq. (18) with respect to ${\mathbf{x}}$ derives the following:

19 $$\begin{aligned} \frac{{\partial {\mathbf{f}}\left( {{\mathbf{x}}_{es}} \right) }}{{\partial {\mathbf{x}}}} = {\mathbf{0}}. \end{aligned}$$

Using the chain rule of the composite function, Eq. (19) can be written as

20 $$\begin{aligned} \frac{{\partial {\mathbf{f}}\left( {{\mathbf{x}}_{es}} \right) }}{{\partial {\mathbf{x}}_{es}}}\frac{{\partial {\mathbf{x}}_{es}}}{{\partial {\mathbf{x}}}} = {\mathbf{0}}. \end{aligned}$$

From the definition of equilibrium space given by Eq. (8), we have

21 $$\begin{aligned} \frac{{\partial {\mathbf{x}}_{es}}}{{\partial {\mathbf{x}}}} = \frac{\partial }{{\partial {\mathbf{x}}}}\left\{ {{\mathbf{Tx}} + \left( {{{\mathbf{I}}} - {{\mathbf{T}}}} \right) {{\mathbf{\chi }}_{es}}} \right\} = {{\mathbf{T}}}. \end{aligned}$$

By noting that

22 $$\begin{aligned} \frac{{\partial {\mathbf{f}}\left( {{\mathbf{x}}_{es}} \right) }}{{\partial {\mathbf{x}}_{es}}} = {\left. {\frac{{\partial {\mathbf{f}}\left( {\mathbf{x}} \right) }}{{\partial {\mathbf{x}}}}} \right| _{{\mathbf{x}} = {\mathbf{x}}_{es}}} = D{\mathbf{f}}\left( {{\mathbf{x}}_{es}} \right) , \end{aligned}$$

substituting Eqs. (21) and (22) into Eq. (20) allows for the derivation of Eq. (17). $\square$

Then the following gives the proof of theorem 2.

#### Proof

By differentiating the function ${{\mathbf{f}}_{es}}$ given by Eq. (11) with respect to ${\mathbf{x}}$, we get

23 $$\begin{aligned} \frac{{\partial {{\mathbf{f}}_{es}}\left( {\mathbf{x}} \right) }}{{\partial {\mathbf{x}}}} = \frac{\partial }{{\partial {\mathbf{x}}}}\left\{ {D{\mathbf{f}}\left( {{\mathbf{x}}_{es}} \right) \left( {{\mathbf{x}} - {\mathbf{x}}_{es}} \right) } \right\} = \frac{{\partial \left\{ {D{\mathbf{f}}\left( {{\mathbf{x}}_{es}} \right) } \right\} }}{{\partial {\mathbf{x}}}}\left( {{\mathbf{x}} - {\mathbf{x}}_{es}} \right) + \;D{\mathbf{f}}\left( {{\mathbf{x}}_{es}} \right) \frac{{\partial \left( {{\mathbf{x}} - {\mathbf{x}}_{es}} \right) }}{{\partial {\mathbf{x}}}}. \end{aligned}$$

By using the result of Lemma 1 and Eq. (21), we can deduce that

24 $$\begin{aligned} D{\mathbf{f}}\left( {{\mathbf{x}}_{es}} \right) \frac{{\partial \left( {{\mathbf{x}} - {\mathbf{x}}_{es}} \right) }}{{\partial {\mathbf{x}}}} = D{\mathbf{f}}\left( {{\mathbf{x}}_{es}} \right) \left( {{{\mathbf{I}}} - {{\mathbf{T}}}} \right) = D{\mathbf{f}}\left( {{\mathbf{x}}_{es}} \right) . \end{aligned}$$

Substituting Eq. (24) into Eq. (23) gives

25 $$\begin{aligned} \frac{{\partial {{\mathbf{f}}_{es}}\left( {\mathbf{x}} \right) }}{{\partial {\mathbf{x}}}} = \frac{{\partial \left\{ {D{\mathbf{f}}\left( {{\mathbf{x}}_{es}} \right) } \right\} }}{{\partial {\mathbf{x}}}}\left( {{\mathbf{x}} - {\mathbf{x}}_{es}} \right) + \;D{\mathbf{f}}\left( {{\mathbf{x}}_{es}} \right) . \end{aligned}$$

By substituting ${\mathbf{x}}_{es}$ for ${\mathbf{x}}$ in Eq. (25), we can derive

26 $$\begin{aligned} {\left. {\frac{{\partial {{\mathbf{f}}_{es}}\left( {\mathbf{x}} \right) }}{{\partial {\mathbf{x}}}}} \right| _{{\mathbf{x}} = {\mathbf{x}}_{es}}} = D{\mathbf{f}}\left( {{\mathbf{x}}_{es}} \right) . \end{aligned}$$

By Noting that

27 $$\begin{aligned} {\left. {\frac{{\partial {{\mathbf{f}}_{es}}\left( {\mathbf{x}} \right) }}{{\partial {\mathbf{x}}}}} \right| _{{\mathbf{x}} = {\mathbf{x}}_{es}}} = D{{\mathbf{f}}_{es}}\left( {{\mathbf{x}}_{es}} \right) , \end{aligned}$$

we can rewrite Eq. (26) as

28 $$\begin{aligned} D{{\mathbf{f}}_{es}}\left( {{\mathbf{x}}_{es}} \right) = D{\mathbf{f}}\left( {{\mathbf{x}}_{es}} \right) . \end{aligned}$$

An arbitrary point ${\mathbf{x}}_{ep} \in {\mathbf{X}}_{es}$ is an equilibrium point of system (1), which satisfies Eq. (28), i.e.,

29 $$\begin{aligned} D{{\mathbf{f}}_{es}}\left( {\mathbf{x}}_{ep} \right) = D{\mathbf{f}}\left( {\mathbf{x}}_{ep} \right) . \end{aligned}$$

Thus, following the Lyapunov’s indirect method presented in “Equilibrium point and Lyapunov’ linearization” section, the stabilities of ${\mathbf{x}}_{es}$ in the original system (1) and the pseudo linearized system (11) are locally identical. $\square$

#### Remark 5

The results for the equilibrium point and equilibrium state presented in “Equilibrium point and Lyapunov’ linearization” and “Equilibrium space and pseudo linearization” sections are also available for the system with a control input, i.e.,

30 $$\begin{aligned} \dot{{\mathbf{x}}}\left( t \right) = {\mathbf{f}}\left( {{\mathbf{x}}\left( t \right) ,\,\,{{\mathbf{u}}}\left( t \right) } \right) . \end{aligned}$$

## Discrete-time model of CMG system based on pseudo linearization

**Figure 1:**
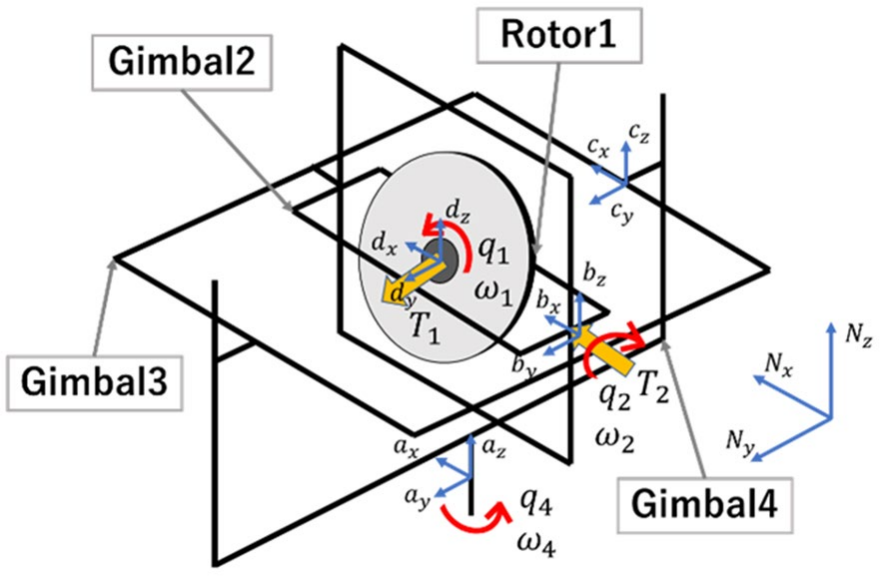
Structure of the CMG system.

The CMG is considered as an example for applying the proposed equilibrium point and pseudo linearization. The CMG is an application of the gyro effect and is often used as an attitude control actuator for artificial satellites and spacecraft. It is mainly composed of four rigid bodies, as shown in Fig. 1. Rotor 1 (flywheel) rotates at high speeds to accumulate angular momentum, and by tilting gimbals 2, 3, and 4, the rotational force of rotor 1 can be output to any other rotational axis. In this study, to describe the formula more explicitly and without loss of generality, we consider the 3-axis drive CMG model using gimbal 3, which is fixed.

### State space model of CMG

Let ${J_{ix}},\,\,{J_{iy}},\,\,{J_{iz}}$ be the moments of inertia of the rigid body *i* with respect to the fixed *x*, *y*, and *z* axes, respectively; let $q_i$ and $\omega _i$ be the relative angle and relative angular velocity of the rigid body *i* with respect to the rigid body $i+1$ ($i = 1,\,\,2,\,\,3$), respectively; ${q_4}$ and $\omega _4$ are the relative rotation angle and angular velocity of gimbal 4 with respect to the inertial coordinate system, respectively; and $\tau _1$ and $\tau _2$ are the external torques used to control the rotation of rotor 1 and gimbal 2, respectively.

A state space model of the CMG system can be derived in the form of Eq. (30) by using the Euler–Lagrange equation of motion^29^, where the system function is given by

31 $$\begin{aligned} {\mathbf{f}}\left( {{\mathbf{x}},{{\mathbf{u}}}} \right) = \left[ {\begin{array}{*{20}{c}} {{\omega _2}}\\ {\begin{array}{*{20}{c}} {{\omega _4}}\\ {{f_1}\left( {\mathbf{x}} \right) + {g_1}\left( {\mathbf{x}} \right) {\tau _1}}\\ {{f_2}\left( {\mathbf{x}} \right) + {g_2}\left( {\mathbf{x}} \right) {\tau _2}} \end{array}}\\ {{f_4}\left( {\mathbf{x}} \right) + {g_4}\left( {\mathbf{x}} \right) {\tau _1}} \end{array}} \right] . \end{aligned}$$

In Eq. (31), $${\mathbf{x}} = {\left[ {\begin{array}{*{20}{c}} {{q_2}}&{\begin{array}{*{20}{c}} {{q_4}}&{{\omega _1}}&{{\omega _2}} \end{array}}&{{\omega _4}} \end{array}} \right] ^T}$$ and $${{\mathbf{u}}} = {\left[ {\begin{array}{*{20}{c}} {{\tau _1}}&{{\tau _2}} \end{array}} \right] ^T}$$ are the system state and system input, respectively. It should be noted that the rotation angle $q_1$ does not appear in the equation of motion of the CMG; thus, it is not considered within the state variables. In addition, $q_1$ can be calculated by integrating the angular velocity $\omega _1$. Functions ${f_i}\left( {\mathbf{x}} \right)$ and ${g_i}\left( {\mathbf{x}} \right)$ are given as below:

32 $$\begin{aligned} {f_1}\left( {\mathbf{x}} \right)&= \frac{2}{Q}\left\{ R{\omega _2}S_{{q_2}}^2C_{{q_2}}^3\left( {R{\omega _4}S_{{q_2}}^1 - {J_{1x}}{\omega _1}} \right) + {\omega _2}C_{{q_2}}^2\left( \left( {A + P} \right) R{\omega _4} - {J_{1x}}\left( {P - B} \right) {\omega _1}S_{{q_2}}^1 \right. \right. \nonumber \\&\quad - \left. \left. \left( {A + 2P} \right) R{\omega _4}S_{{q_2}}^2 + {J_{1x}}R{\omega _1}S_{{q_2}}^3 \right) \right\} , \end{aligned}$$

33 $$\begin{aligned} {g_1}\left( {\mathbf{x}} \right)&= \frac{2}{Q}\left( {PB - \frac{{{R^2}}}{4}S_{{q_2}}^2} \right) , \end{aligned}$$

34 $$\begin{aligned} {f_2}\left( {\mathbf{x}} \right)&= \frac{1}{A}{\omega _4}C_{{q_2}}^1\left( {R{\omega _4}S_{{q_2}}^1 - {J_{1x}}{\omega _1}} \right) , \end{aligned}$$

35 $$\begin{aligned} {g_2}\left( {\mathbf{x}} \right)&= \frac{1}{A}, \end{aligned}$$

36 $$\begin{aligned} {f_4}\left( {\mathbf{x}} \right)&= \frac{2}{Q}\left\{ {J_{1x}}\left( {{J_{2x}} + {J_{3x}}} \right) {\omega _2}\left( {2{\omega _1}C_{{q_2}}^1 - {\omega _4}} \right) - {\omega _2}{\omega _4}\left( {{J_{2x}} - {J_{1z}} - {J_{2z}}} \right) \left( {{J_{2x}} + 2{J_{3x}} + {J_{1z}} + {J_{2z}} + RC_{{q_2}}^2} \right) \right\} , \end{aligned}$$

37 $$\begin{aligned} {g_4}\left( {\mathbf{x}} \right)&= \frac{2}{Q}\left( {{J_{3x}} + {J_{1z}} + {J_{2z}}} \right) S_{{q_2}}^1, \end{aligned}$$

where

38 $$\begin{aligned} A&= {J_{1y}} + {J_{2y}}, \end{aligned}$$

39 $$\begin{aligned} B&= {J_{3z}} + \left( {{J_{1x}} + {J_{2x}}} \right) C_{{q_2}}^2 + \left( {{J_{1z}} + {J_{2z}}} \right) S_{{q_2}}^2, \end{aligned}$$

40 $$\begin{aligned} P&= {J_{3x}} + \left( {{J_{1x}} + {J_{2x}}} \right) C_{{q_2}}^2 + \left( {{J_{1z}} + {J_{2z}}} \right) S_{{q_2}}^2, \end{aligned}$$

41 $$\begin{aligned} Q&= \left( {{J_{2x}} + {J_{1z}} + {J_{2z}}} \right) \left( {{J_{3x}} + {J_{3z}} + {J_{4z}}} \right) + {J_{2x}}\left( {{J_{1z}} + {J_{2z}}} \right) + {J_{3x}}\left( {{J_{3z}} + {J_{4z}}} \right) - \left( {{J_{2x}} - {J_{1z}} - {J_{2z}}} \right) \left( {{J_{3x}} - {J_{3z}} - {J_{4z}}} \right) C_{2{q_2}}^1, \end{aligned}$$

42 $$\begin{aligned} R&= {J_{1x}} + {J_{2x}} - {J_{1z}} - {J_{3z}}. \end{aligned}$$

In the above equation, $S_{\theta }^{i}$ and $C_{\theta }^{i}$ are defined by

43 $$\begin{aligned} S_{\theta }^{i}&= {\sin ^i}\theta , \end{aligned}$$

44 $$\begin{aligned} C_{\theta }^{i}&= {\cos ^i}\theta . \end{aligned}$$

### Discrete-time model for CMG system based on pseudo linearization

When the system input is zero, i.e., $${{\mathbf{u}}} = {\left[ {\begin{array}{*{20}{c}}{{\tau _1}}&{{\tau _2}}\end{array}} \right] ^T} = {\mathbf{0}}$$, following Eq. (31), we have

45 $$\begin{aligned} {\mathbf{f}}\left( {{\mathbf{x}},{\mathbf{0}}} \right) = {\left[ {\begin{array}{*{20}{c}} {{\omega _2}}&{{\omega _4}}&{{f_1}\left( {\mathbf{x}} \right) }&{{f_2}\left( {\mathbf{x}} \right) }&{{f_4}\left( {\mathbf{x}} \right) } \end{array}} \right] ^T}. \end{aligned}$$

It should be noted that substituting ${\omega _2} = {\omega _4} = 0$ into Eqs. (32), (34), and (36) yields

46 $$\begin{aligned} {f_1}\left( {\mathbf{x}} \right) = {f_2}\left( {\mathbf{x}} \right) = {f_4}\left( {\mathbf{x}} \right) = 0. \end{aligned}$$

regardless of the values of $q_2$, $q_4$, and $\omega _1$. The equilibrium space of the CMG system can be written as

47 $$\begin{aligned} {\mathbf{x}}_{es}\left( t \right) = {\mathbf{Tx}}\left( t \right) + \left( {{{\mathbf{I}}} - {{\mathbf{T}}}} \right) {{\mathbf{\chi }}_{es}}, \end{aligned}$$

where

48 $$\begin{aligned} {{\mathbf{T}}} = \left[ {\begin{array}{*{20}{c}} 1&{}\quad 0&{}\quad 0&{}\quad 0&{}\quad 0\\ 0&{}\quad 1&{}\quad 0&{}\quad 0&{}\quad 0\\ 0&{}\quad 0&{}\quad 1&{}\quad 0&{}\quad 0\\ 0&{}\quad 0&{}\quad 0&{}\quad 0&{}\quad 0\\ 0&{}\quad 0&{}\quad 0&{}\quad 0&{}\quad 0 \end{array}} \right] \end{aligned}$$

49 $$\begin{aligned} {{\mathbf{\chi }}_{es}} = {\left[ {\begin{array}{*{20}{c}} 0&\quad 0&\quad 0&\quad 0&\quad 0 \end{array}} \right] ^T}. \end{aligned}$$

Equation (47) can also be written as

50 $$\begin{aligned} {\mathbf{x}}_{es}\left( t \right) = {\left[ {\begin{array}{*{20}{c}} {{q_2}}&\quad {\begin{array}{*{20}{c}} {{q_4}}&\quad {{\omega _1}}&\quad 0 \end{array}}&\quad 0 \end{array}} \right] ^T}. \end{aligned}$$

The pseudo linearization of the CMG system about the above equilibrium state is given by

51 $$\begin{aligned} \dot{{\mathbf{x}}} = {\left. {\frac{{\partial {\mathbf{f}}\left( {{\mathbf{x}},\,{\mathbf{0}}} \right) }}{{\partial {\mathbf{x}}}}} \right| _{{\mathbf{x}} - {\mathbf{x}}_{es}}}\left( {{\mathbf{x}} - {\mathbf{x}}_{es}} \right) + \left[ {\begin{array}{*{20}{c}} 0&{}\quad 0\\ 0&{}\quad 0\\ {{g_1}\left( {{\mathbf{x}}_{es}} \right) }&{}0\\ 0&{}{{g_2}\left( {{\mathbf{x}}_{es}} \right) }\\ {{g_1}\left( {{\mathbf{x}}_{es}} \right) }&{}0 \end{array}} \right] {{\mathbf{u}}}. \end{aligned}$$

The system described by Eq. (51) has the form of a linear system, i.e.,

52 $$\begin{aligned} \dot{{\mathbf{x}}} = {{\mathbf{A}}}\left( {{\mathbf{x}} - {\mathbf{x}}_{es}} \right) + {\mathbf{Bu}}, \end{aligned}$$

which has an exact discrete-time model as detailed in^30^

53 $$\begin{aligned} \delta {{\mathbf{x}}_k} = \int _0^T {{e^{A\tau }}d\tau } \left( {{{\mathbf{A}}}\left( {{{\mathbf{x}}_k} - {{\mathbf{x}}_{es,\,k}}} \right) + {{\mathbf{B}}}{{{\mathbf{u}}}_k}} \right) , \end{aligned}$$

where *T* is a sampling interval, and $\delta$ is a delta operator defined by

54 $$\begin{aligned} \delta {{\mathbf{x}}_k} = \frac{{{{\mathbf{x}}_{k + 1}} - {{\mathbf{x}}_k}}}{T}. \end{aligned}$$

#### Remark 6

A discrete-time model of the system (30) derived by the forward-difference method is given by

55 $$\begin{aligned} \delta {{\mathbf{x}}_k} = {\mathbf{f}}\left( {{{\mathbf{x}}_k},\,\,{{{\mathbf{u}}}_k}} \right) . \end{aligned}$$

## Simulations

Simulations were carried out using MATLAB/Simulink to compare the responses of the discrete-time models for the CMG system derived by the conventional linearized discretization method (CL), the forward-difference method (FD), the conventional pseudo linear representation method (CPL), and the proposed equilibrium space-based pseudo linearization method (ESPL) with the original nonlinear continuous-time response (CT). The moments of inertia of the CMG used in the simulations is given by Table 1^24^.

**Table 1:** Moments of inertia of the CMG.

	Rigid body 1	Rigid body 2	Rigid body 3	Rigid body 4
$J_x$ [kg m$^{2}$]	0.0273	0.0281	0.0178	–
$J_y$ [kg m$^{2}$]	0.0148	0.0124	0.0119	–
$J_z$ [kg m$^{2}$]	0.0273	0.0188	0.0297	0.0693

**Figure 2:**
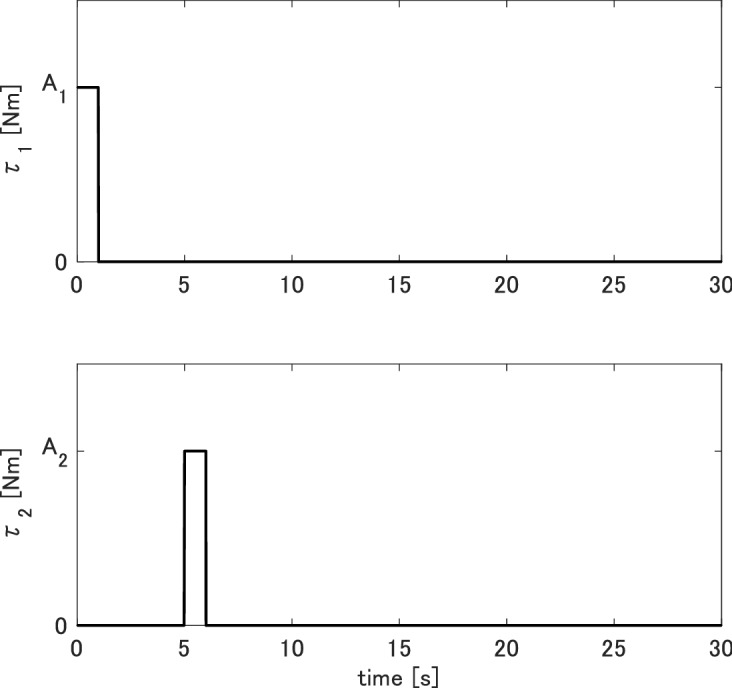
Waveform of the input torques.

In all the simulations, the initial value of the system was set to zero, i.e., ${{\mathbf{x}}_0} = {\mathbf{0}}$, and the simulation time was 30s. The input torque, $\tau _1$, used to rotate the inner wheel, and that, $\tau _2$, used to tilt gimbal 2 to produce the gyro effect, are given by the following pulse signals

56 $$\begin{aligned} \left\{ {\begin{array}{*{20}{c}} {{\tau _1} = {A_1}\left( {{u_s}\left( t \right) - {u_s}\left( {t - 1} \right) } \right) \,\,\,\,\,\,\,\,}\\ {{\tau _2} = {A_2}\left( {{u_s}\left( {t - 5} \right) - {u_s}\left( {t - 6} \right) } \right) ,} \end{array}} \right. \end{aligned}$$

where $\tau _1$ is an unit step function, and $A_1$ and $A_2$ are amplitudes of $\tau _1$ and $\tau _2$, respectively. The waveform of the input torques is shown by Fig. 2. When $A_1$ is large and $A_2$ is small, the CMG is strongly stable. By contrast, when $A_1$ is small and $A_2$is large, the CMG is weakly stable. The accuracy of a discrete-time model, in general, depends on the value of the sampling interval *T*. In the simulations, we investigated the responses of the discrete-time models with various values of the amplitudes $A_1$, $A_2$, and the sampling period *T*. The parameters are summarized in Table 2.

**Table 2:** Varying parameters used for simulations.

	Case 1	Case 2	Case 3	Case 4
*T* [s]	0.0001	0.0001	0.001	0.001
$A_1$ [Nm]	1.0	2.0	1.0	2.0
$A_2$ [Nm]	1.0	1.0	1.0	1.0

**Figure 3:**
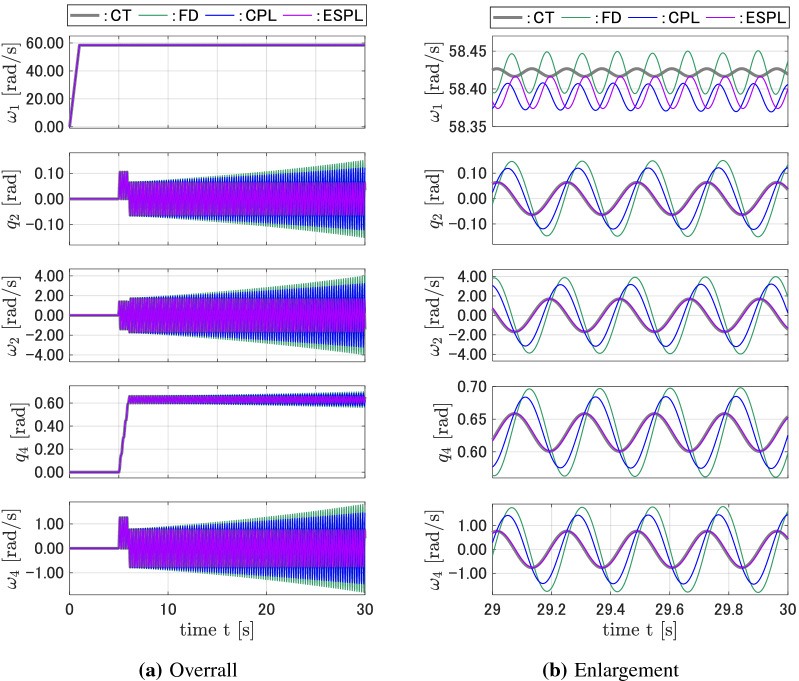
Responses of the CT, FD, CPL, and ESPL models for case 1.

**Figure 4:**
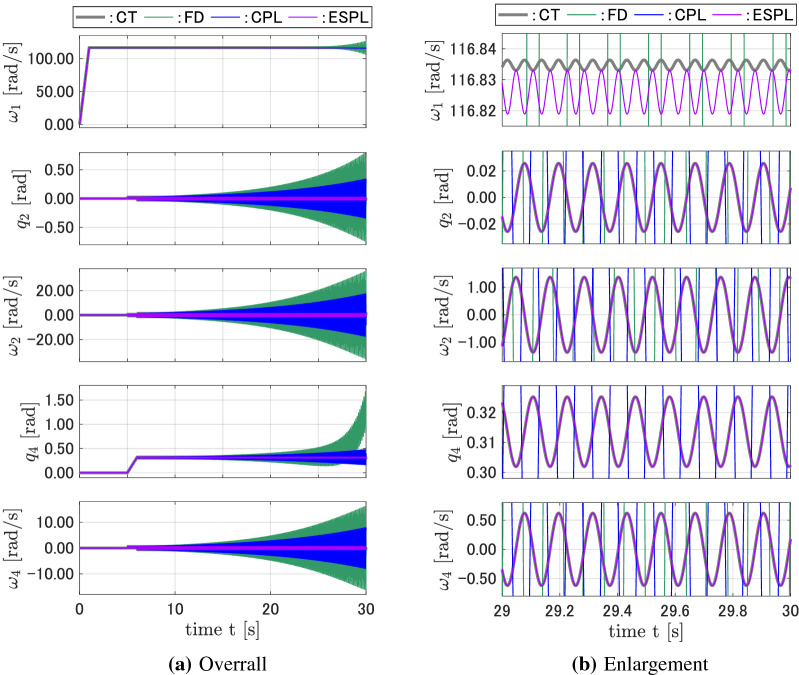
Responses of the CT, FD, CPL, and ESPL models for case 2.

**Figure 5:**
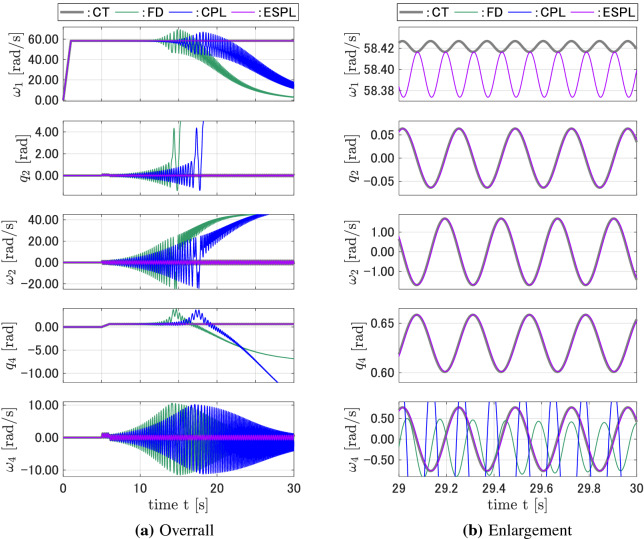
Responses of the CT, FD, CPL, and ESPL models for case 3.

**Figure 6:**
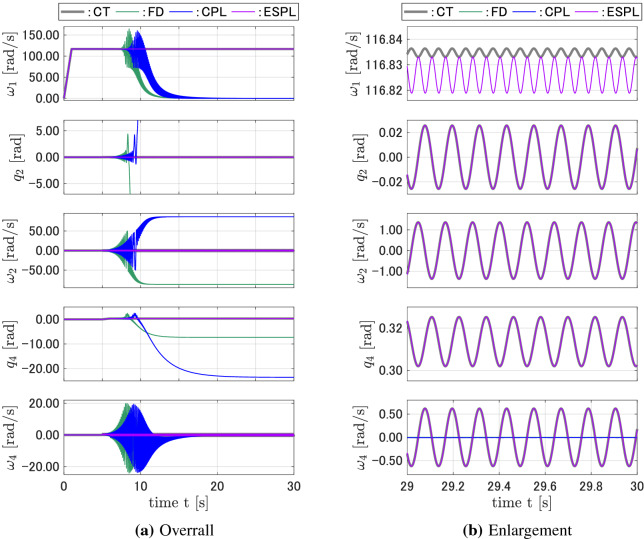
Responses of the CT, FD, CPL, and ESPL models for case 4.

Figure 3a shows the responses of the discrete-time models compared with that of the continuous-time model for $${A_1} = {A_2} = $$1.0N m and $$T = $$ 0.0001 s. Figure 3b is an enlargement of Fig. 3a for $$29 \le t \le 30$$. While the responses for $$q_2$$, $$\omega _2$$, $$q_4$$, and $$\omega _4$$ of the FD and CPL discrete-time models tended to diverge from the continuous-time responses when the time *t* increased, the proposed ESPL discrete-time model yielded responses that are close to that of the CT model. The response for $$q_1$$ of all three discrete-time models had high-frequency vibrations whose amplitude and phase essentially differed from that of the CT model. Figure 4 shows the responses for the system shown in Fig. 3, but with $${A_1} = $$ 2.0N m. In this case, the states of the CMG vibrate with a higher frequency. The responses of the FD and CPL models started to diverge sooner, while the ESPL yielded accurate responses compared with that of the CT model. Figures 5 and 6 show the responses for the system, where the torque amplitudes $$A_1$$ and $$A_2$$ have the same values as that shown in Figs. 3 and 4, respectively, but with a sampling interval of $$T = $$0.001 s, which is 10 times that shown in Figs. 3 and 4. While the proposed discrete-time ESPL model provided responses that are exactly close to that of the continuous-time CT model with a large sampling interval, the discrete-time FD and CPL did not retain the features, and their responses were significantly different from that of the CT model.

**Figure 7:**
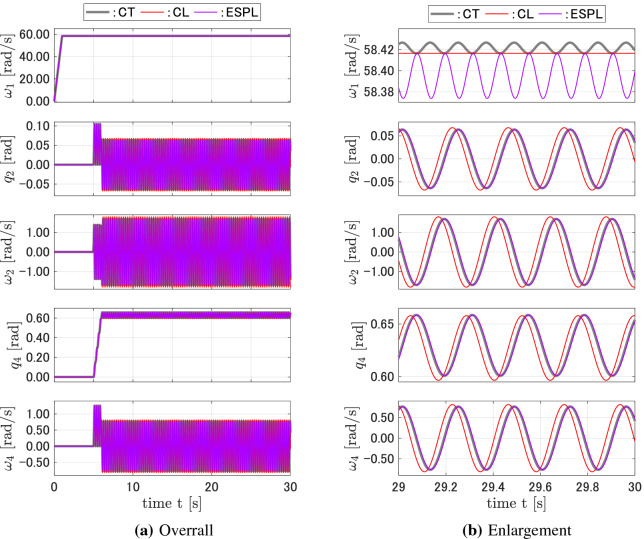
Responses of the CT, CL, and ESPL models for the (relatively) exact equilibrium.

**Figure 8:**
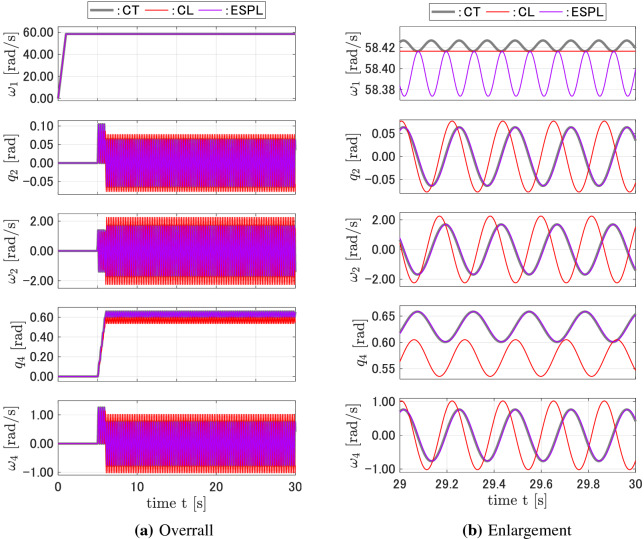
Responses of the CT, CL, and ESPL models for the equilibrium point with error.

Another simulation was carried out to compare the ESPL model derived from the proposed pseudo linearization based on the state equilibrium and the CL model derived from the conventional linearization about the equilibrium point. Since the CMG system has an infinite number of equilibrium points, the steady state of the CMG can be considered as a candidate for the equilibrium point from which the conventional linearization is taken. However, the steady state of the CMG depends on the input torques and cannot be calculated analytically. Furthermore, high-frequency vibrations remain within the steady-state. These issues become significant when considering the conventional linearization about the equilibrium point. In this study, the continuous-time model was first simulated, and subsequently, its steady state, whose high-frequency vibrations were filtered out, was used as the equilibrium point for the linearization. Consider a system with the same parameters as shown in Fig. 5. The equilibrium point for this case is estimated from the continuous-time responses as $${\mathbf{x}}_{ep} = {\left[ {\begin{array}{*{20}{c}}0&{\begin{array}{*{20}{c}}{0.638}&\quad {29.2}&\quad 0\end{array}}&0\end{array}} \right] ^T}$$. Figure 7 shows the responses of the CL and the proposed ESPL discrete-time models, as compared to that of the continuous-time CT model. Although the equilibrium point was estimated as accurately as possible, there were differences between the characteristics and the responses of the CL and CT models. The vibration of $\omega _1$ in the CT model was not reproduced within the CL model. When the equilibrium point was estimated with an error, for example, $10\%$ of $\omega _1$ as compared to the case shown in Fig. 7, i.e., $${\mathbf{x}}_{ep} = {\left[ {\begin{array}{*{20}{c}}0&{\begin{array}{*{20}{c}}{0.638}&\quad {31.1}&\quad 0\end{array}}&\quad 0\end{array}} \right] ^T}$$, the responses of the CL model were different to that of the CT model, not only in amplitude but also in the frequency of the vibrations (Fig. 8). The proposed ESPL model yielded accurate responses and did not require equilibrium point to be estimated.

## Conclusion

In this paper, a new concept of equilibrium space is proposed, which is an extension of the concept of an equilibrium point in space. The well-known Lyapunov’s indirect theorem is a useful tool for investigating the stability of a single equilibrium point. However, for systems with an infinite number of equilibrium points, investigating all the equilibrium points separately is not realistic. The concept of an equilibrium space can therefore be considered as an effort to bridge this gap when considering the equilibrium point. Although this concept is equivalent to the manifold of equilibria proposed by scholars before, the definition of equilibrium space proposed in this study is expected to bring it nearer to applications in engineering’s problems. In the sense of Lyapunov’s linearization method, this paper proposes a pseudo linearization, by which we can derive a nonlinear system presented in the linear form. The equilibrium state of this pseudo linearization and its stability are shown to be the same as that of the original nonlinear system. To demonstrate the potential applications, the proposed pseudo linearization was used to derive a discrete-time model for the CMG system from a nonlinear continuous-time model. Simulation results showed that the discrete-time model derived using the proposed pseudo linearization yielded responses that were closer to that of the continuous-time model than the discrete-time models derived by the well-known forward-difference, the conventional pseudo linear representation, and the linearization about the equilibrium point methods, even with a large sampling interval. Investigating applications of the pseudo linearization based on the equilibrium state for system analysis and control design will be considered as the next step.

## Data Availability

The datasets used and/or analysed during the current study available from the corresponding author on reasonable request.
